# Effect of lateral internal sphincterotomy in patients undergoing excisional hemorrhoidectomy

**DOI:** 10.1097/MD.0000000000011820

**Published:** 2018-08-10

**Authors:** Wei-Guo Wang, Wen-Zhu Lu, Chun-Mei Yang, Ke-Qiang Yu, Hong-Bo He

**Affiliations:** a Department of Integrated Traditional Chinese and Western Medicine, West China Hospital, Sichuan University; b Department of Integrated Traditional Chinese and Western Medicine, Cheng Du Shang Jin Nan Fu Hospital, Chengdu, Sichuan Province, China.

**Keywords:** excisional hemorrhoidectomy, hemorrhoids, lateral internal sphincterotomy, postoperative pain

## Abstract

**Background:**

Excisional hemorrhoidectomy (EH) is the major surgical option for high-grade symptomatic hemorrhoids, but it has some shortcomings, especially postoperative pain. This study was performed to assess the effect of lateral internal sphincterotomy (LIS) in patients undergoing excisional hemorrhoidectomy.

**Methods:**

A systematic literature search (Medline, Embase, Cochrane Library, Science Citation Index, Science Direct, Springer Link, Ovid Journals, and EBSCO) was performed to identify all eligible articles. Randomized controlled trials (RCTs) published until July 7, 2017 comparing EH combined with LIS (experimental group) with EH only (control group) were eligible for inclusion. The primary outcome of interest was postoperative pain.

**Results:**

Ten RCTs involving 1560 patients were identified for inclusion. The pooled analysis revealed that patients undergoing EH and LIS were associated with lower pain score [standardized mean difference (SMD), −0.75; 95% confidence interval (CI), −1.14 to −0.36; z = 3.76; *P* = .0002] and resting anal pressure [odds ratio (OR), −17.19; 95% CI, −25.66 to −8.72; z = 3.98; *P* < .0001], and lower incidence of anal stricture (OR, 0.12; 95% CI, 0.03–0.53; z = 2.85; *P* = .004). However, the differences of urinary retention, bleeding and length of hospital stay were similar between the 2 methods.

**Conclusion:**

Our meta-analysis suggests that LIS effectively relieves postoperative pain and reduces patient's postoperative analgesic requirements. LIS also reduces the incidence of anal stenosis but increases the incidence of fecal incontinence.

## 1 Introduction

A high prevalence of hemorrhoids (up to 40%) is found during screening colonoscopy in the general population,^[1]^ and 44.7% of patients with hemorrhoids are symptomatic and require interventions.^[2]^ And excisional hemorrhoidectomy (EH) is the most effective treatment for high-grade symptomatic hemorrhoids with a 2% medium-term recurrence rate and a 10% long-term recurrence rate.^[2]^

However, EH has some shortcomings, especially postoperative pain. The cause of this postoperative pain is multifactorial. One contributory factor may be spasm of the internal anal sphincter (IAS), which is exposed and impinged after EH.^[3,4]^ Therefore, different methods and techniques have been employed to overcome this inevitable problem.

Lateral internal sphincterotomy (LIS) is a widely used adjunct treatment following EH. Its advocators have stated that LIS can abolish spasm of the IAS and subsequently relieve postoperative pain. However, this view has not been consistently supported by recent clinical trials. Some researchers have reported limited ability of LIS to relieve postoperative pain and increased incidence of other complications, such as bleeding and fecal incontinence. Few large-sample prospective clinical trials involving this problem have been performed to date, and the precise role of LIS following EH remains controversial.

The primary objective of this systematic review was to analyze existing randomized controlled trials (RCTs) regarding the effect of LIS on postoperative pain in patients undergoing EH and perform a meta-analysis of postoperative pain, other complications, and length of hospital stay.

## 2 Methods

### 2.1 Selection of studies

A systematic literature search (Medline, Embase, Cochrane Library, Science Citation Index, Science Direct, Springer Link, Ovid Journals, and EBSCO) was performed to identify all eligible articles. RCTs published until 7 July 2017 comparing hemorrhoidectomy combined with LIS (experimental group) with hemorrhoidectomy only (control group) were eligible for inclusion. The following Medical Subject Headings were used: “hemorrhoids,” “pain, postoperative,” “sphincterotomy,” and “hemorrhoidectomy.” Their combinations or similar headings were also searched, including “hemorrhoid,” “postoperative pain,” “internal sphincterotomy,” “lateral sphincterotomy,” and “Milligan-Morgan.” A personal search was also performed using the reference lists of the retrieved relevant articles and reviews to identify additional trials and ensure that all potential studies were included.

### 2.2 Inclusion and exclusion criteria

All included trials were required to fulfill the following criteria: designed as an RCT, involved humans and were published in English, provided clear documentation of “LIS and EH” as the treatment in the experimental group, reported the treatment as “EH” in the control group, and when 2 or multiple studies were published by the same institution and/or authors, either the higher-quality study or the most recent trial was included in the meta-analysis. Studies were excluded if it was impossible to extract the appropriate data, such as abstracts, case reports, letters, reviews, and commentaries; there was no control group; the number of cases was <20; and the follow-up duration was <2 weeks.

### 2.3 Study eligibility assessment

Two authors (W-GW and W-ZL) independently scanned the title and abstract of each publication to identify potentially eligible studies. The full articles were then obtained for detailed evaluation. Any disagreement in the selection process was resolved through consensus. If this failed, a third author (H-BH) adjudicated.

### 2.4 Outcome evaluation

The following outcomes were compared among the included studies: postoperative pain, postoperative analgesic requirement, fecal incontinence, anal stricture, urinary retention, bleeding, and duration of hospital stay.

The primary outcome of interest was postoperative pain. For the purpose of comparing pain scores, the highest pain intensity during the first 24 hours after the operation was compared among the included trials. All pain scores or numeric rating scale scores were converted to a scale of 0 to 10.

### 2.5 Data extraction

Two authors (C-MY and K-QY) independently extracted data from all eligible studies using standardized forms. The following data were extracted from each study: first author, country, study period, study design, participant characteristics, technical results, definition of clinical results, and outcomes during the follow-up period. Any disagreements were resolved using the same method mentioned above. We also attempted to contact the authors of all eligible RCTs if any missing data or inaccurate information was encountered.

### 2.6 Quality assessment

The Cochrane Risk of Bias tool was used to assess the quality of the RCTs.^[5]^ Any disagreement was resolved through consensus.

### 2.7 Ethics and dissemination

In this study, the extracted data was collected from published studies. Based on this, it did not require ethical approval. The results of this study will eventually be published in a peer-reviewed journal.

### 2.8 Statistical analysis

The meta-analysis was performed in line with the recommendations of the Cochrane Collaboration and the Quality of Reporting of Meta-analyses guidelines.^[6,7]^ Statistical analysis of dichotomous variables was carried out using the odds ratio (OR) as the summary statistic, while continuous variables were analyzed using the standardized mean difference (SMD); both were reported with the 95% confidence interval (CI).^[7]^ The OR represented the odds of an adverse event occurring in the LIS + EH group versus the EH-only group, and it was considered statistically significant at *P* < .05 if the 95% CI did not include the value 1. The SMD summarized the differences in continuous variables between the 2 groups, and it was considered statistically significant at *P* < .05 if the 95% CI did not cross the value 0. Heterogeneity between studies was measured using χ^2^ and *I*^2^, and *I*^2^ > 50% was considered statistically significant. Either a fixed-effects model or random-effects model was applied to calculate the pooled effect based on the heterogeneity. However, a random-effects model was used first to assess the heterogeneity. A sensitivity analysis, with 1 study removed from the meta-analysis at a time, was performed to assess the stability of the results. All statistical analyses were conducted using the statistical software Review Manager (version 5.3).

## 3 Results

### 3.1 Selection of studies

**Figure 1:**
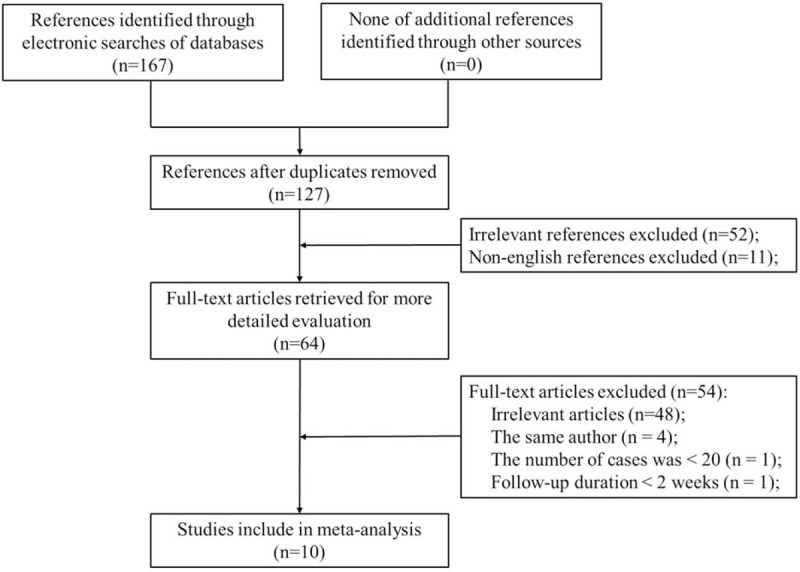
Flow chart showing the search strategy used to identify studies.

**Figure 2:**
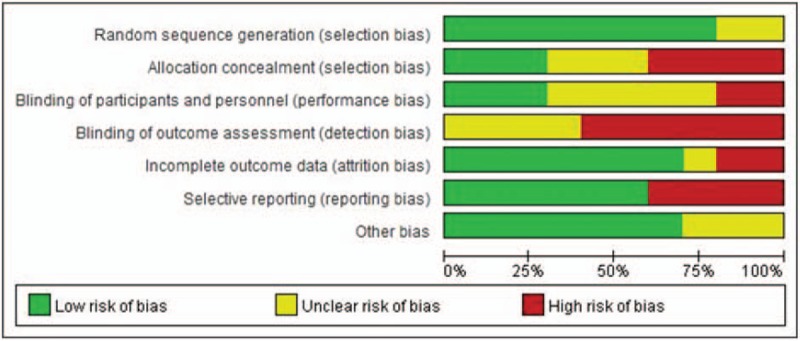
Summary of bias of the included studies.

In total, 167 articles were obtained using the above-described search strategy. After the screening process, however, 103 articles were excluded. Of the remaining 64 articles, 54 were excluded and 10 RCTs were finally included in the review (Fig. 1).^[4,8–16]^ The quality of the RCTs is shown in Figure 2.

The most important characteristics of the pooled trials are summarized in Table 1. The effect of LIS in patients undergoing EH was compared among a total of 1560 patients with grade II to IV hemorrhoids.

**Table 1:**
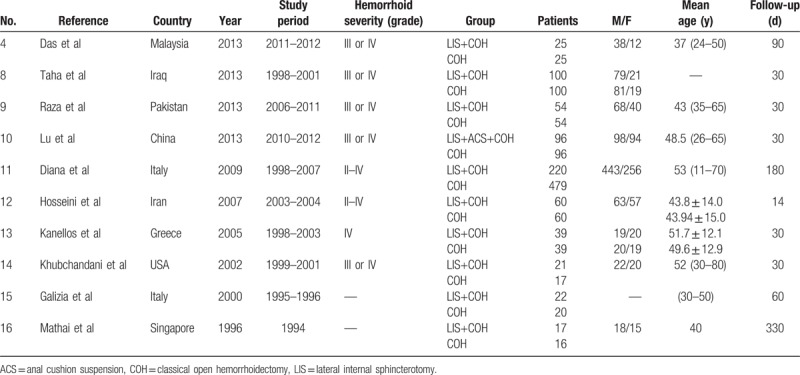
Characteristics of the included studies.

### 3.2 Postoperative pain

The technical characteristics of pain management are shown in Table 2. The methods of anesthesia differed among the included studies: general anesthesia was used in 4 studies,^[4,11,13,16]^ locoregional anesthesia was used in 3 studies,^[10,14,15]^ and the type of anesthesia was not reported in 3 studies.^[8,9,12]^ Seven of the 10 included studies added injectable or oral analgesics to manage pain after the operation.

**Table 2:**
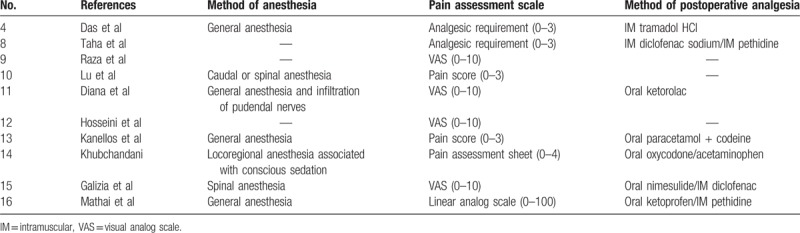
Technical characteristics of pain management in the included studies.

The methods of pain assessment also differed among the included studies: 4 studies used a visual analog scale ranging from 0 to 10 points,^[9,11,12,15]^ 4 studies used a pain score ranging from 0 to 3 points,^[4,8,10,13]^ 1 study used a pain assessment sheet ranging from 0 to 4 points,^[14]^ and 1 study used a linear analog scale ranging from 0 to 100 points.^[16]^

**Figure 3:**
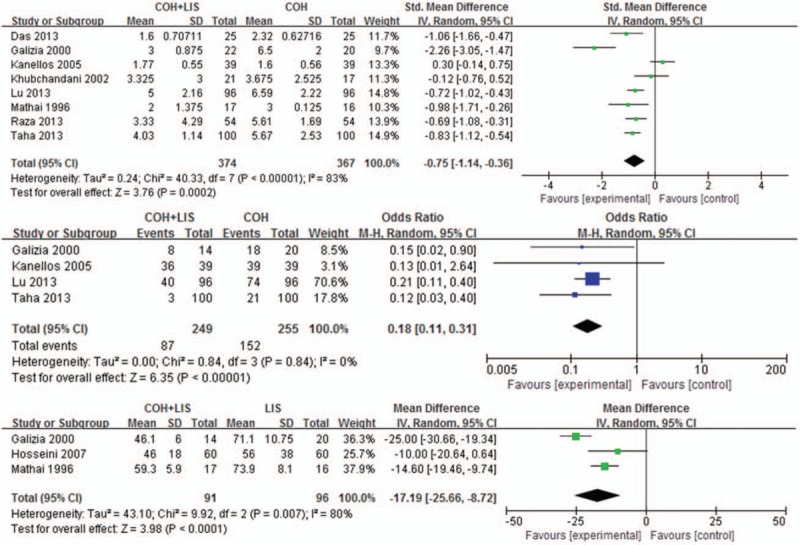
Forest plots of (A) postoperative pain scores, (B) patients requiring analgesia, and (C) resting anal pressure. Odds ratios are shown with 95% confidence intervals.

Pain score data was obtained from 8 studies;^[4,8–10,13–16]^ the data from 2 studies was excluded because they only reported the mean pain score.^[11,12]^ There was significant heterogeneity among these studies (χ^2^ = 106.24, df = 7, *P* < .00001, *I*^2^ = 83%). In the random-effects model (standardized mean difference, −0.75; 95% CI, −1.14 to −0.36; z = 3.76; *P* = .0002), the pain score was significantly lower in the experimental group of patients undergoing EH and LIS than in the control group of patients undergoing EH only (Fig. 3A).

The method of postoperative analgesia was described in 7 studies: 2 studies used intramuscular analgesics,^[4,8]^ whereas 4 studies used oral analgesics.^[11,13–16]^ Furthermore, 4 studies reported the detailed percentage of patients who were administered analgesia. The meta-analysis showed that this percentage was significantly lower in the experimental group of patients undergoing EH and LIS than in the control group of patients undergoing EH only (OR, 0.18; 95% CI, 0.10–0.30; z = 6.49; *P* < .00001) (Fig. 3B).^[8,10,13,15]^

Postoperative anorectal manometry was performed in 3 studies.^[12,15,16]^ A significant decrease in the resting anal pressure in patients undergoing EH and LIS was confirmed by our meta-analysis (OR, −17.19; 95% CI, −25.66 to −8.72; z = 3.98; *P* < .0001) (Fig. 3C).

### 3.3 Anal stricture

**Figure 4:**
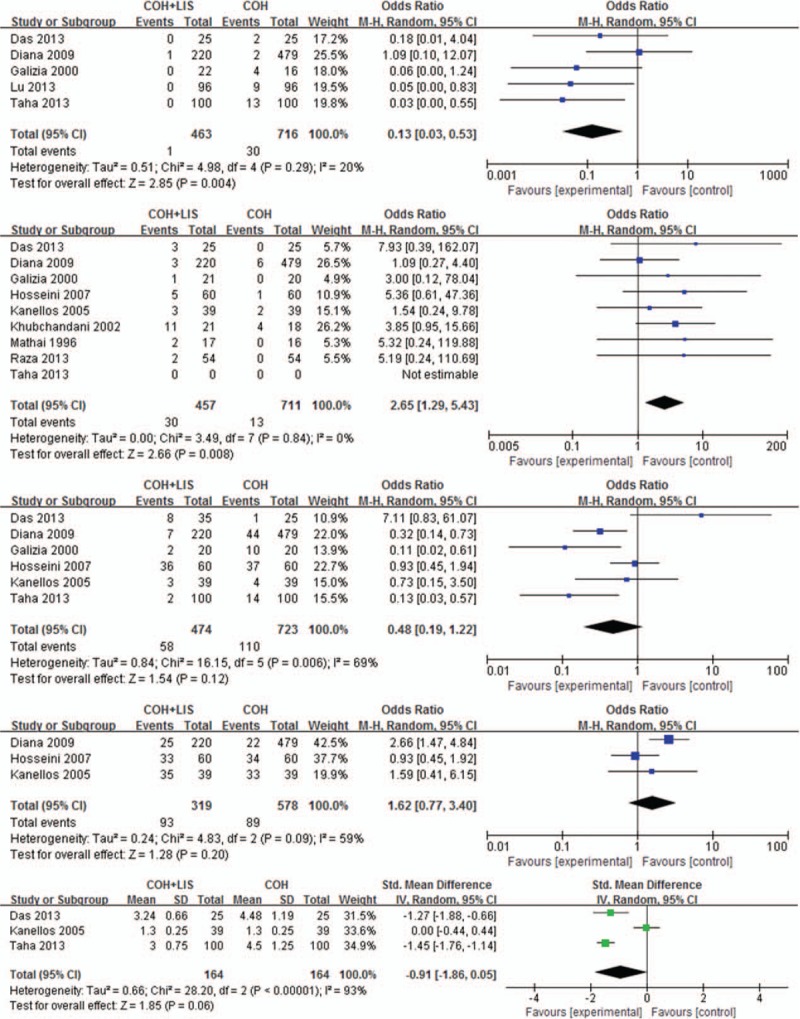
Forest plots of (A) anal stricture, (B) fecal incontinence, (C) urinary retention, (D) bleeding, and (E) hospital stay. Odds ratios are shown with 95% confidence intervals.

Five studies reported the incidence of anal stricture.^[4,8,10,11,15]^ These results were combined by the random-effects model and revealed significantly lower incidence in the experimental than control group (OR, 0.12; 95% CI, 0.03–0.53; z = 2.85; *P* = 0.004) (Fig. 4A).

### 3.4 Fecal incontinence

Eight studies reported the incidence of fecal incontinence.^[4,9,11–16]^ In the pooled analysis, this complication developed in 6.6% (30/457) of patients in the experimental group but in only 1.8% (13/711) of patients in the control group. The meta-analysis showed a significant difference between the 2 groups (OR, 2.65; 95% CI, 1.29–5.43; z = 2.66; *P* = .08) (Fig. 4B).

### 3.5 Urinary retention

Six studies reported the incidence of urinary retention.^[4,8,11–13,15]^ There was significant heterogeneity among the included studies (χ^2^ = 16.15, df = 5, *P* = .006, *I*^2^ = 69%). In the random-effects model, the incidence of urinary retention was similar between the 2 groups (OR, 0.48; 95% CI, 0.19–1.22; z = 1.54; *P* = .12) (Fig. 4C).

### 3.6 Bleeding

The incidence of bleeding was described in 3 trials.^[11–13]^ Diana et al^[11]^ reported a higher incidence of bleeding in the experimental than control group (11.90% vs. 6.83%, respectively), but the other 2 studies did not find a significant difference between the 2 groups.^[12,13]^ Furthermore, the meta-analysis did not show a significant difference in bleeding (OR, 1.62; 95% CI, 0.77–3.34; z = 1.28; *P* = .020) (Fig. 4D).

### 3.7 Hospital stay

The hospital stay was reported in 3 studies.^[4,8,13]^ Das et al^[4]^ and Taha^[8]^ reported a shorter hospital stay for patients who underwent EH with LIS. However, Kanellos et al^[13]^ found a similar hospital stay between the 2 groups. Furthermore, because of the significant heterogeneity among the studies [tau^2^ = 0.66, χ^2^ = 28.20, df = 2 (*P* < .00001), *I*^2^ = 93%], the hospital stay showed no significant difference between the 2 groups in the random-effects model (OR, −0.91; 95% CI, −1.86–0.05; z = 1.85; *P* = .06) (Fig. 4E).

### 3.8 Sensitivity analysis

Because of the insignificant results in the sensitivity analysis, the stability of the meta-analysis was proved when each study was deleted from the pooled analysis.

### 3.9 Publication bias

**Figure 5:**
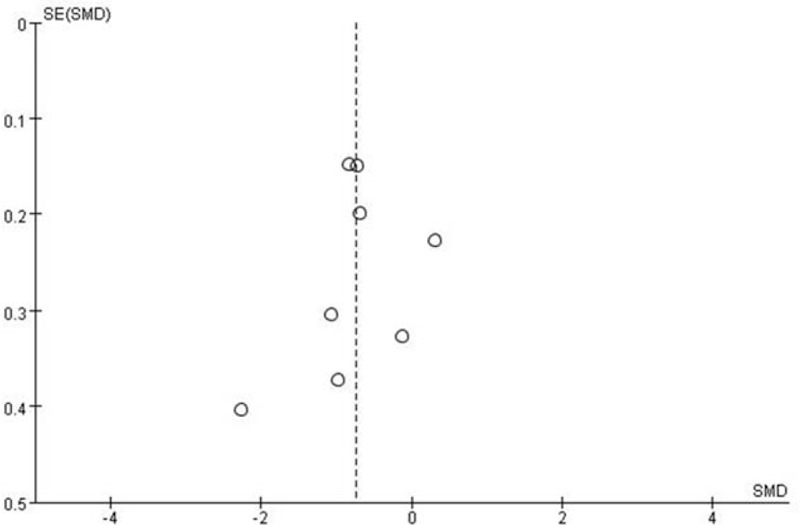
Funnel plot of pain scores for publication bias.

A funnel plot of the incidence of postoperative pain is shown in Figure 5. The symmetrical distribution indicated no evidence of publication bias among the included studies.

## 4 Discussion

To the best of our knowledge, this is the first systematic review and meta-analysis of RCTs evaluating the effect of LIS in patients undergoing EH.

Postoperative pain is an unpleasant physiological and emotional experience following surgical damage.^[17]^ As one of the most important complaints after EH is postoperative pain that may be associated with several secondary complications such as difficult defecation, urinary retention, and a prolonged hospital stay.^[18]^ An accurate and comprehensive assessment is essential to effectively monitor the severity and duration of postoperative pain and ensure proper management of postoperative pain. Patient self-reporting, which is based on trust, cartoons, or imaginary data^[19]^ and reveals the subjectivity of postoperative pain, is currently a popular observational measurement for researchers.

As for postoperative pain scores, our result showed that there was significant heterogeneity among included RCTs (χ2 = 106.24, df = 7, *P* < .00001, I^2^ = 83%). The heterogeneity presented the current research status of all included RCTs. Currently, more than 25 types of pain scales were applicable to target population with different characteristics: neonates, infants, children, adolescents, adults, older person, and persons whose communication is impaired.^[20]^ So inherent subjectivity and interindividual variation are basic characteristics of the postoperative pain scale scores among include RCTs.^[21]^ Furthermore, the technical characteristics of pain management (Table 2) showed that observational measurements of postoperative pain, methods of anesthesia, and postoperative pain managements differed among the included RCTs. Seven RCTs added injectable or oral analgesics to manage pain after surgery, which would affect patient's feeling after surgery. Therefore, the heterogeneity was potentially affected not only by different pain scales but also by various methods of pain management. And owning to the long time span of included RCTs (ranging from 2002 to 2013), the heterogeneity of these RCTs was significantly. We found lower postoperative pain scores and analgesic requirements in patients who underwent EH and LIS. The heterogeneities of existing RCTs affected our finding that the combination of EH with LIS potentially relieves postoperative pain significantly more than EH only. It advocated further large samples, multicenter RCT to get the more reliable and conceivable conclusion.

Actually, the current most widely accepted opinion is that spasm of the IAS is one of the main causes of postoperative pain after EH. The IAS is an involuntary muscle that contributes about 55% of the resting anal pressure.^[12]^ Anorectal manometry is the direct and objective method for assessing the anal musculature tone, rectal compliance, and anorectal sensation and verifying the integrity of the rectoanal inhibitory reflex.^[22]^ A high resting anal pressure has been documented in patients with hemorrhoids, especially younger patients.^[12,23]^ An increased pressure not only causes spasm of the IAS but also blocks the normal blood and lymph circulation of the anus.^[24]^ Our meta-analysis proved that LIS effectively decreases the resting anal pressure after EH.

Furthermore, due to decrease in anal pressure, LIS is believed to prevent anal stricture, a rare but serious complication after EH. Anal stricture develops in only 5% of patients after EH but always results in serious outcomes such as anal pain, constipation, obstipation, and bleeding.^[25]^ The main cause of anal stricture is overzealous excision of large areas of the anoderm and hemorrhoidal rectal mucosa from the lining of the anal canal.^[26]^ And the performance of LIS can create adequate mucocutaneous bridges between adjacent wounds. Furthermore, LIS has been proven to be a simple, safe, and adequate intervention for functional stenosis and mild, low anal stricture (anal diameter of 1.0–1.5 cm).^[27]^ For severe anal stricture, however, a formal anoplasty is more efficient than LIS to treat the loss of anal tissue.

Nevertheless, our results show that decrease in anal pressure causes another complication after EH, namely fecal incontinence, which always occurs with an anal pressure of <40 mm Hg.^[28]^ This is one of the most frequent complaints following EH (6.6% of patients).^[29]^ But postoperative anorectal manometry is not the routine test after EH. Only 3 RCTs performed postoperative anorectal manometry and reported a significant decrease in the resting anal pressure in patients undergoing EH and LIS. The drawback of existing RCTs is the short of long-term manometric study. LIS is also performed to resolve anal fissure. The change of anal pressure had been evaluated following LIS in patients with anal fissure. In 2005, Edward et al performed a prospective study that included 50 patients with anal fissure and 12 healthy volunteers.^[30]^ In their studies, the resting anal pressure was significantly decreased after LIS and gradually increased within 1 year after surgery but still remained significantly lower than before surgery.^[30]^ Other similar results were also reported. Lewis et al^[31]^ reported the incidence of fecal incontinence is 17% in their patients after LIS and 2/3 of fecal incontinence were only temporary. Khubchandani et al reported the incidence of fecal incontinence is 22% in their patients and 35.1% of these patients was classified grade 1.^[32]^ These results reported the long-term, dynamic, minor, temporary, and acceptable change of anal pressure after LIS.^[33]^ Some studies have assessed the efficacy of several drugs, such as nitroglycerin^[34]^ and botulin toxin,^[35]^ for temporary relaxation of the IAS to help wound healing while avoiding permanent damage to the IAS. The long-term advantage of these drugs is limited. But in general, the changes of IAS and anal pressure regarding the effect of LIS in patients undergoing EH needed to be proved in further long-term manometric study.

Urinary retention is another common complication after EH.^[36]^ In the present meta-analysis, the incidence of urinary retention was 14.0%. However, the difference was not significant between patients who did and did not undergo LIS following EH. The exact mechanism of urinary retention remains unclear; it might be caused by dysfunction of the detrusor muscle and the detrusor of the anal canal. Furthermore, many risk factors for urinary retention have been documented in previous studies: advanced age, sex, anesthesia methods, severity of hemorrhoids, perioperative fluid administration, hospital stay duration, and others.^[36,37]^ Therefore, sufficient evidence with which to prove the effect of LIS on urinary retention is lacking.

The incidence of postoperative bleeding was also similar between patients who did and did not undergo LIS following EH. Most patients develop mild early postoperative bleeding after EH, especially during defecation. However, such bleeding is always temporary and resolves without intervention. The similar incidence of postoperative bleeding between the 2 groups proves that LIS is a safe additional intervention in patients undergoing EH.

Because of the effective decrease in postoperative pain, performing LIS within EH may also shorten patients’ hospital stay and allow them to return to their normal activities of daily life. However, only 3 studies assessed the hospital stay, and the decrease in the hospital stay was not significant in our meta-analysis. Different centers usually apply different hospital discharge criteria, which may have contributed to the heterogeneity of the hospital stay among the studies in this meta-analysis.

Several limitations of this meta-analysis should be taken into account. First, only English-language articles and no unpublished trials were included in this meta-analysis. Second, various pain assessment scales were used, and a unified definition of postoperative pain (the main outcome of this meta-analysis) was lacking. Finally, the sample size of most included studies was relatively small.

## 5 Conclusions

LIS, an additional intervention to EH, effectively relieves postoperative pain and reduces patients’ postoperative analgesic requirements. LIS reduces the incidence of anal stenosis but increases the incidence of fecal incontinence. The effect of LIS in patients undergoing excisional EH should be assessed with well-designed, high-quality, large-sample RCTs in the future.

## Author contributions

W-GW and H-BH designed the study, W-GW and W-ZL collected related data, W-GW performed most of the research, W-GW wrote the manuscript, H-BH offered valuable suggestions, and C-MY and K-QY edited the manuscript.

**Data curation:** Wen-Zhu Lu.

**Formal analysis:** Wei-Guo Wang.

**Investigation:** Chun-Mei Yang.

**Methodology:** Wen-Zhu Lu.

**Project administration:** Ke-Qiang Yu.

**Validation:** Ke-Qiang Yu.

**Visualization:** Chun-Mei Yang.

**Writing – original draft:** Wei-Guo Wang.

**Writing – review & editing:** Hong-Bo He.
